# 两种兔抗人胸腺细胞免疫球蛋白给药方案同胞全相合造血干细胞移植治疗恶性血液病的临床研究

**DOI:** 10.3760/cma.j.issn.0253-2727.2023.08.008

**Published:** 2023-08

**Authors:** 基山 都, 海涛 汪, 立萍 窦, 楠 王, 菲 李, 香淑 金, 代红 刘

**Affiliations:** 中国人民解放军总医院、解放军总医院第五医学中心血液病医学部，北京 100853 Chinese PLA General Hospital, Department of Hematology in the Fifth Medical Center of PLA General Hospital, Beijing 100853, China

**Keywords:** 造血干细胞移植, 抗胸腺细胞球蛋白, 药代动力学, 复发, Allogeneic hematopoietic stem cell transplantation, Antithymocyte globulin, Pharmacokinetics, Relapsed

## Abstract

**目的:**

比较两种兔抗人胸腺细胞免疫球蛋白（rATG）预处理给药方案同胞全相合造血干细胞移植（MSD-HSCT）治疗恶性血液病的疗效。

**方法:**

纳入2020年10月至2022年4月在解放军总医院第一医学中心血液科接受含rATG 5 mg/kg预处理MSD-HSCT的32例患者，按照rATG给药方案分为4天给药组（4d-rATG组，16例）和2天给药组（2d-rATG组，16例），4d-rATG组移植前5～2 d给药，2d-rATG组移植前5、4 d给药。对两组患者临床资料进行回顾性分析，比较两组移植结局、移植前4 d至移植后28 d患者血清活性抗胸腺细胞球蛋白（ATG）浓度及移植后30、60、90 d淋巴细胞亚群重建情况。

**结果:**

①4d-rATG组、2d-rATG组移植后100 d急性移植物抗宿主病（GVHD）的累积发生率分别为25.0％（95％*CI* 7.8％～47.2％）、18.8％（95％*CI* 4.6％～40.2％）（*P*＝0.605），1年慢性GVHD累积发生率分别为25.9％（95％*CI* 8.0％～48.6％）、21.8％（95％*CI* 5.2％～45.7％）（*P*＝0.896），移植后1年累积复发率分别为37.5％（95％*CI* 18.9％～65.1％）、14.6％（95％*CI* 3.6％～46.0％）（*P*＝0.135），1年总生存率分别为75.0％（95％*CI* 46.3％～89.8％）、100％（*P*＝0.062）。②4d-rATG组、2d-rATG组患者总血清活性ATG浓度-时间曲线下面积（AUC）分别为36.11、35.89 UE/ml·d（*P*＝0.984），4d-rATG组移植后AUC高于2d-rATG组（20.76 UE/ml·d对15.95 UE/ml·d，*P*＝0.047）。③4d-rATG组、2d-rATG组移植后3个月CD8^+^T淋巴细胞计数分别为852（680～1968）个/µl、623（611～764）个/µl（*P*＝0.037）。

**结论:**

4d-rATG方案与2d-rATG方案具有相近的GVHD预防作用；2d-rATG组CD8^+^T淋巴细胞重建优于4d-rATG组，可能与其移植后活性ATG浓度较低有关。

## Abstract (en)

**Objective:**

To compare the effects of two administration time strategies for rabbit antihuman thymocyte immunoglobulin（rATG）of 5mg/kg total dose in matched sibling donor hematopoietic stem cell transplantation（MSD-HSCT）.

**Methods:**

This study retrospectively analyzed the clinical data of 32 patients who received MSD-HSCT with 5 mg/kg rATG conditioning regimen at the Department of Hematology of the First Medical Center of the People's Liberation Army General Hospital from October 2020 to April 2022. The patients were classified into two groups: the 4d-rATG group（16 cases）, who received antithymocyte globulin（ATG）from day −5 to day −2, and the 2d-rATG group（16 cases）, who received ATG from day −5 to day −4. Between the two groups, the transplantation outcomes, serum concentrations of active antithymocyte globulin（ATG）in patients from −4 days to 28 days after graft infusion（+28 days）, and the reconstitution of lymphocyte subsets on days +30, +60, and +90 were compared.

**Results:**

The cumulative incidences of acute graft-versus-host disease at 100 days after graft infusion were 25.0％（95％*CI* 7.8％–47.2％）and 18.8％（95％*CI* 4.6％–40.2％）（*P*＝0.605）in the 4d-rATG group and 2d-rATG group, respectively. The 1-year cumulative incidences of chronic graft-versus-host disease were 25.9％（95％*CI* 8.0％–48.6％）and 21.8％（95％*CI* 5.2％–45.7％）（*P*＝0.896）. The 1-year cumulative incidence of relapse was 37.5％（95％*CI* 18.9％–65.1％）and 14.6％（95％*CI* 3.6％–46.0％）（*P*＝0.135）, and the 1-year probabilities of overall survival were 75.0％（95％CI 46.3％–89.8％）and 100％（*P*＝0.062）. The total area under the curve（AUC）of serum active ATG was 36.11 UE/ml·d and 35.89 UE/ml·d in the 4d-rATG and 2d-rATG groups, respectively（*P*＝0.984）. The AUC was higher in the 4d-rATG group than that in the 2d-rATG group（20.76 UE/ml·d *vs* 15.95 UE/ml·d, *P*＝0.047）. Three months after graft infusion, the average absolute count of CD8^+^ T lymphocytes in the 4d-rATG group was lower than that in the 2d-rATG group（623 cells/µl *vs* 852 cells/µl, *P*＝0.037）.

**Conclusion:**

The efficiencies of GVHD prophylaxis in MSD-PBSCT receiving 4d-ATG regimen and the 2d-rATG regimen were found to be similar. The reconstruction of CD8^+^T lymphocytes in the 2d-rATG group was better than that in the 4d-rATG group, which is related to the lower AUC of active ATG after transplantation.

异基因造血干细胞移植（allo-HSCT）是治疗血液系统恶性疾病的有效方法，其中同胞全合供者造血干细胞移植（MSD-HSCT）迄今仍是首选移植模式。随着外周血造血干细胞移植体系的推广，移植后慢性移植物抗宿主病（GVHD）发生率较高的问题越来越受到重视[1]–[2]。抗胸腺细胞球蛋白（ATG）是一种多克隆球蛋白，可在体内抑制T细胞活性，对GVHD有预防作用[3]。在同胞全相合造血干细胞移植（MSD-HSCT）患者的预处理方案中加入ATG可以降低cGVHD的发生率[4]。但ATG在降低GVHD发生率的同时也可能抑制移植后免疫重建，带来复发率增加的风险，风险高低与ATG暴露量相关[5]–[6]。ATG暴露量的影响因素主要包括ATG给药剂量以及距离移植物输注时间[7]。有研究显示使用减低剂量兔ATG的患者免疫重建优于使用常规剂量rATG的患者，感染及复发风险也较低[8]–[9]。ATG给药时间对患者的影响目前研究较少，Lindemans等[10]在儿童无关供者移植中对比了移植前9 d（−9 d）～−5 d使用兔ATG 10 mg/kg与−5 d～0 d使用兔ATG 10 mg/kg两组患者的预后及免疫重建情况，早期使用兔ATG的患者T细胞免疫重建优于晚期使用兔ATG患者，但也有较晚期使用ATG致急性GVHD发生率增高的问题。目前，ATG的最佳给药时机仍不明确。本中心通过分析两种兔抗人胸腺细胞免疫球蛋白（rATG）给药方案MSD-HSCT治疗恶性血液病患者近期疗效、血清活性ATG（ATG中能与人体淋巴细胞结合的成分）浓度及移植后淋巴细胞亚群重建情况，探索rATG暴露时间对移植后早期免疫重建及其临床转归的影响。

## 病例与方法

1. 病例：纳入2020年10月至2022年4月期间在解放军总医院第一医学中心血液科接受MSD-HSCT的32例恶性血液病患者。入选病例满足以下条件：①患者年龄16～65岁、供者年龄<68岁；②诊断为急性白血病（AL）或骨髓增生异常综合征（MDS）；③具有MSD-HSCT的指征。按照rATG给药方案分为4天给药组（4d-rATG组，16例）和2天给药组（2d-rATG组，16例）。本研究得到解放军总医院伦理委员会的批准，并获得患者的知情同意。

2. 治疗方案：所有患者均接受清髓预处理方案：2例急性淋巴细胞性白血病（ALL）患者接受全身放射治疗（TBI）方案，其余患者接受改良白消安（Bu）/环磷酰胺（Cy）的预处理方案，具体预处理用药及GVHD和感染预防方案参照文献[11]。rATG给药时间：4d-rATG组移植前5 d（−5 d）～−2 d，2d-rATG组−5 d、−4 d，两组rATG总量均为5 mg/kg。

3. 流式细胞术检测外周血活性ATG浓度：本研究将造血干细胞回输日定义为0 d。分别于−4 d、−3 d、−2 d、−1 d、0 d、+7 d、+14 d、+21 d、+28 d采集患者外周血标本进行活性ATG浓度检测[12]。以1 mg ATG中所含的活性ATG量为1个等效单位（UE），以药-时曲线下面积（AUC）代表活性ATG的有效暴露，计量单位为UE/ml·d。通过梯形面积法计算移植前（−4 d～0 d）、移植后（0 d～+28 d）活性ATG的AUC，−4 d～+28 d的AUC定义为总AUC。

3. 流式细胞术检测淋巴细胞亚群：于移植后1、2、3个月收集患者外周血样本，应用流式细胞术进行淋巴细胞亚群检测。

4. 随访：采用查阅住院/门诊病例和电话联系进行随访。截止日期为2022年10月15日。移植后中位随访时间为14.0（5.7～24.5）个月，4d-rATG组20.6（14.3～24.5）个月，2d-rATG组10.7（5.7～13.6）个月。

5. 定义：急性GVHD的诊断和分级依据改良的Glucksberg分级标准[13]；慢性GVHD的诊断和分级根据美国国立卫生研究院（NIH）的标准[14]；巨细胞病毒（CMV）与EB病毒（EBV）再激活定义为CMV/EBV DNA拷贝数连续2次≥1×10^3^/ml或单次≥1×10^4^/ml；复发定义为骨髓/外周血白血病细胞≥5％或发生髓外白血病；总生存（OS）期定义为干细胞回输至随访截止或任何原因死亡的时间。

6. 统计学处理：采用EZR统计软件和SPSS 26.0软件进行统计学分析。分类资料的组间比较采用卡方检验。符合正态分布的计量资料数据采用*t*检验，不服从正态分布的计量资料则采用秩和检验。采用竞争风险模型计算EBV/CMV再激活、GVHD和复发的累积发生率。病毒再激活的竞争事件是无病毒再激活的死亡，GVHD的竞争事件包括复发和死亡。组间病毒再激活率、GVHD、复发和NRM的累积发生率差异比较用Gray检验。OS用Kaplan-Meier进行估计，单因素和多因素分析采用Cox比例风险回归分析。ATG使用方案及单因素分析中*P*≤0.2的变量全部纳入多因素分析，*P*<0.05被认为具有统计学意义。

## 结果

1. 患者临床特征：本研究纳入32例MSD-HSCT患者。4d-rATG组16例，骨髓增生异常综合征（MDS）2例，急性髓系白血病（AML）8例，急性淋巴细胞白血病（ALL）7例。2d-rATG组16例，AML 9例，ALL 7例。4d-rATG组移植前第1次完全缓解期（CR1）患者数量高于2d-rATG组（*P*＝0.049），其余两组患者临床基线特征差异均无统计学意义（*P*均>0.05）。详见表1。

**表1:** 两种rATG给药方案同胞全相合造血干细胞移植恶性血液病患者临床基线特征比较

临床特征	2d-rATG组（16例）	4d-rATG组（16例）	统计量	*P*值
移植年龄［岁，*M*（范围）］	44（19~61）	37（19~60）	*z*＝−0.868	0.386
年龄分组［例（％）］			*χ*^2^＝2.032	0.154
≤40岁	7（43.8）	11（68.8）		
>40岁	9（56.3）	5（31.3）		
性别［例（％）］			*χ*^2^＝2.032	0.154
男	7（43.8）	11（68.8）		
女	9（56.3）	5（31.3）		
患者体重［kg，*M*（范围）］	60.5（38.6~75.5）	69.0（49.6~85.6）	*z*＝−1.960	0.050
诊断［例（％）］			*χ*^2^＝2.136	0.344
AML	9（56.3）	8（50.0）		
MDS	0	2（12.5）		
ALL	7（43.8）	6（37.5）		
移植前状态［例（％）］			*χ*^2^＝3.865	0.049
CR1	9（56.3）	14（87.5）		
未达CR1	7（43.8）	2（12.5）		
DRI评分［例（％）］			*χ*^2^＝2.667	0.264
低危	0	2（12.5）		
中危	4（25.0）	2（12.5）		
高危	12（75.0）	12（75.0）		
HCT-CI评分［例（％）］			*χ*^2^＝2.091	0.554
0分	8（50.0）	8（50.0）		
1分	6（37.5）	11（31.3）		
2分	1（6.3）	3（18.8）		
3分	1（6.3）	0		
预处理方案［例（％）］			*χ*^2^＝2.133	0.144
Bu-Cy方案	14（87.5）	16（100）		
TBI方案	2（12.5）	0		
供者年龄［岁，*M*（范围）］	39.5（9～59）	42（14～53）	*z*＝−0.698	0.485
供患者血型［例（％）］			*χ*^2^＝0.783	0.853
相合	9（56.3）	7（43.8）		
主要不相合	4（25.0）	4（25.0）		
次要不相合	2（12.5）	3（18.8）		
双向不相合	1（6.3）	2（12.5）		
供患者性别组合［例（％）］			*χ*^2^＝4.521	0.210
男供男	4（25.0）	5（31.3）		
女供女	7（43.8）	3（18.8）		
男供女	4（25.0）	3（18.8）		
女供男	1（6.3）	5（31.3）		
移植物来源［例（％）］			*χ*^2^＝0.237	0.626
外周血	13（81.3）	14（87.5）		
外周血+骨髓	3（18.8）	2（12.5）		
单个核细胞输注量［×10^8^/kg，*M*（范围）］	14.25（8.14～29.40）	13.68（7.05～33.45）	*z*＝−0.377	0.706
CD34^+^细胞输注量［×10^6^/kg，*M*（范围）］	4.48（1.42～13.07）	3.98（2.07～8.60）	*z*＝−0.572	0.572

注：rATG：兔抗人胸腺细胞免疫球蛋白；AML：急性髓系白血病；ALL：急性淋巴细胞白血病；MDS：骨髓增生异常综合征；Bu-Cy：白消安+环磷酰胺；TBI：全身放射治疗；HCT-CI：造血干细胞移植共患病指数；DRI：疾病危险度积分；CR1：第一次完全缓解。2d-rATG组：rATG于移植前5、4 d给药，总量5 mg/kg；4d-rATG组：rATG于移植前5~2 d给药，总量5 mg/kg

2. 造血重建：两组患者在移植后30 d内均获得粒细胞与血小板植入。2d-rATG组、4d-rATG组患者中性粒细胞植入时间分别为11（9～18）d、12（8～29）d（*P*＝0.321），血小板植入时间分别为11.5（8～26）d、13（7～29）d（*P*＝0.970）。

3. GVHD：两组中共有7例患者发生急性GVHD，移植后100 d急性GVHD累积发生率为21.9％（95％*CI* 9.6％～37.2％）。2d-rATG组、4d-rATG组急性GVHD发生率分别为18.8％（95％*CI* 4.6％～40.2％）、25.0％（95％*CI* 7.8％～47.2％）（*P*＝0.605）。两组共有7例患者发生慢性GVHD，1年累积发生率为27.4％（95％*CI* 11.2％～46.5％），2d-rATG组、4d-rATG组移植后1年慢性GVHD累积发生率分别为21.8％（95％*CI* 5.2％～45.7％）、25.9％（95％*CI* 8.0％～48.6％）（*P*＝0.896）。

4. CMV、EBV再激活：2d-rATG组、4d-rATG组移植后180 d EBV再激活率分别为43.8％（95％*CI* 9.6％～23.8％）、56.2％（95％*CI* 15.9％～34.4％（*P*＝0.649），CMV再激活率分别为18.8％（95％*CI* 6.4％～47.5％）、25.0％（95％*CI* 3.6％～10.2％）（*P*＝0.580）。两组均没有发生CMV病或移植后淋巴细胞增殖性疾病。

5. 存活：截至随访终止，2d-rATG组16例（100％）存活，4d-rATG组13例（81.2％）存活。4d-rATG组移植后1年OS率为75％（95％*CI* 46.3％～89.8％），与2d-rATG组（100％）相比无统计学意义（*P*＝0.062）。

复发是患者死亡的主要原因，两组患者共有8例患者复发，中位复发时间为移植后4.4（1.9～6.9）个月，4d-rATG组共有5例复发，其中3例死亡；2d-rATG组共有2例复发，截至随访终点尚带病存活。4d-rATG组、2d-rATG组移植后1年累积复发率分别为37.5％（95％*CI* 18.9％～65.1％）、14.6％（95％*CI* 3.6％～46.0％）（*P*＝0.135）。两组均无非复发死亡病例。

6. 血清活性rATG浓度：两组患者移植前后血清活性rATG浓度见表2。2d-rATG组总血清活性ATG平均浓度（35.89 UE/ml·d）与4d-rATG组（36.11 UE/ml·d）相比差异无统计学意义（*P*＝0.984）；2d-rATG组移植前AUC高于4d-rATG组（19.31 UE/ml·d对14.42 UE/ml·d，*P*＝0.024），移植后AUC低于4d-rATG组（15.95 UE/ml·d对20.76 UE/ml·d，*P*＝0.047）。在针对复发的危险因素分析中发现，移植后中位AUC>18.0 UE/ml·d在单因素与多因素分析中均是复发的危险因素（表3）。

**表2:** 2d-rATG组与4d-rATG组患者MSD-HSCT前后血清活性ATG浓度的比较［UE/ml·d，*x±s*］

组别	−4 d	−3 d	−2 d	−1 d	0 d	+7 d	+14 d	+21 d	+28 d
2d-ATG组	3.33±1.09	7.18±2.47	4.58±1.47	3.82±1.08	2.82±0.67	0.69±0.28	0.20±0.06	0.11±0.05	0.07±0.04
4d-ATG组	1.56±0.65	2.63±0.76	3.47±0.91	5.64±0.92	3.81±0.83	0.86±0.29	0.24±0.08	0.06±0.02	0.05±0.03

**注** rATG：兔抗人胸腺细胞免疫球蛋白；ATG：抗胸腺细胞球蛋白；MSD-HSCT：同胞全相合造血干细胞移植；2d-rATG组：rATG于移植前5、4 d给药，总量5 mg/kg；4d-rATG组：rATG于移植前5～2 d给药，总量5 mg/kg

**表3:** 含rATG预处理同胞全相合造血干细胞移植患者复发相关影响因素的单因素与多因素分析

影响因素	单因素分析		多因素分析	
	*HR*（95％ *CI*）	*P*值	*HR*（95％ *CI*）	*P*值
4d-rATG给药	3.02（0.62~14.6）	0.176	1.24（0.31~4.95）	0.802
移植前AUC>15.5 UE/ml·d	1.02（0.26~3.96）	0.981		
移植后AUC>18.0 UE/ml·d	10.43（1.19~91.56）	0.029	9.44（1.21~73.44）	0.049
患者>40岁	0.36（0.07~1.80）	0.211		
患者为男性	0.72（0.18~2.87）	0.640		
诊断为ALL	0.80（0.21~3.09）	0.758		
移植前疾病状态为非CR1	0.87（0.18~4.18）	0.869		
DRI积分3~4分	0.93（0.21~4.17）	0.926		
HCT-CI评分2~3分	0.51（0.16~1.59）	0.245		
供者>40岁	0.64（0.149~2.74）	0.541		
供者、患者血型不合	0.53（0.13~2.11）	0.384		
女供男组合模式	1.31（0.31~5.58）	0.743		
移植物单个核细胞>8×10^8^/kg	0.30（0.03~2.32）	0.261		
移植物CD34^+^细胞>4×106/kg	0.70（0.16~3.11）	0.664		

**注** 4d-rATG给药：移植前5 d～2 d兔抗人胸腺细胞免疫球蛋白（rATG）给药，总量5 mg/kg；AUC：药-时曲线下面积；ALL：急性淋巴细胞白血病；DRI：疾病危险度积分；HCT-CI：造血干细胞移植共患病指数；CR1：第1次完全缓解

7. 外周血淋巴细胞亚群：2d-rATG组移植后3个月CD8^+^T淋巴细胞计数高于4d-rATG组（*P*＝0.037），其他各时间点CD3^+^ T淋巴细胞、CD4^+^ T淋巴细胞、CD8^+^ T淋巴细胞及NK细胞计数差异无统计学意义（表4）。分析患者移植后3个月的淋巴细胞亚群，移植后AUC<18 UE/ml·d组CD3^+^ T淋巴细胞（*P*＝0.002）、CD4^+^ T淋巴细胞（*P*＝0.045）和CD8^+^ T淋巴细胞（*P*＝0.002）计数均高于AUC>18 UE/ml·d组（表5）。

**表4:** 2d-rATG组与4d-rATG组患者移植后淋巴细胞亚群计数变化［个/µl，*M*（范围）］

组别	例数	CD3^+^细胞			CD4^+^细胞		
		1个月	2个月	3个月	1个月	2个月	3个月
2d-ATG组	16	185（16~692）	712（462~1523）	1032（765~2249）	54（3~141）	69（44~272）	129（40~149）
4d-ATG组	16	189（83~337）	729（615~759）	975（783~1073）	46（16~171）	111（106~132）	143（137~421）

*z*值		−0.234	−0.189	−1.698	−0.078	−0.756	−0.340
*P*值		0.815	0.850	0.089	0.938	0.450	0.734

组别	例数	CD8^+^细胞			NK细胞		
		1个月	2个月	3个月	1个月	2个月	3个月
2d-ATG组	16	103（9~432）	575（377~1093）	852（680~1968）	104（47~173）	215（129~556）	122（77~361）
4d-ATG组	16	129（43~141）	489（448~543）	623（611~764）	89（49~335）	145（83~339）	229（105~239）

*z*值		−0.078	−0.756	−2.087	−0.234	−0.567	−0.243
*P*值		0.938	0.450	0.037	0.815	0.571	0.808

**注** rATG：兔抗人胸腺细胞免疫球蛋白；ATG：抗胸腺细胞球蛋白；MSD-HSCT：同胞全相合造血干细胞移植；2d-rATG组：rATG于移植前5、4 d给药，总量5 mg/kg；4d-rATG组：rATG于移植前5～2 d给药，总量5 mg/kg

**表5:** 移植后AUC<18 UE/ml·d组与移植后AUC<18 UE/ml·d组移植后3个月淋巴细胞亚群分析［个/µl，*M*（范围）］

组别	例数	CD3^+^T淋巴细胞	CD4^+^T淋巴细胞	CD8^+^T淋巴细胞	NK细胞
移植后AUC<18 UE/ml·d组	12	1310（301~2603）	172（131~405）	991（132~2058）	206（88~970）
移植后AUC>18 UE/ml·d组	14	826（329~1436）	129（40~421）	639（197~1271）	127（40~424）

*z*值		−3.138	−2.006	−3.086	−1.492
*P*值		0.002	0.045	0.002	0.136

**注** AUC：药-时曲线下面积

## 讨论

本研究目的是探索ATG给药时间对同胞全合造血干细胞疗效的影响。结果显示，2d-rATG组与4d-rATG组GVHD的预防效果接近，但2d-rATG组患者累积复发率具有降低的趋势，总生存具有改善的趋势。通过检测患者的血清活性ATG浓度并绘制两种方案患者的血清活性ATG浓度-时间曲线，发现4d-rATG组移植后AUC较高，移植后AUC高于中位值18 UE/ml·d的患者有更高的复发风险。2d-rATG组患者移植后3个月CD8^+^T细胞重建优于4d-rATG组患者，移植后AUC<18 UE/ml·d的患者移植后3个月CD3^+^T、CD4^+^T与CD8^+^T细胞重建优于移植后AUC>18 UE/ml·d的患者。

ATG由一组针对多种免疫细胞表位的多克隆抗体组成，可以诱导体内血液和淋巴组织中T细胞的耗竭，通常用于促进植入、预防重度GVHD[4],[11]。ATG在人体内半衰期较长，对移植后供者的淋巴细胞有长时间的作用。ATG作用过强会抑制移植后T细胞的免疫重建[6],[10]，甚至影响具有抗复发作用的T细胞亚群。供者移植物来源的CD8^+^T淋巴细胞和NK细胞可以发挥强效的移植物抗白血病（GVL）效应，有效清除恶性克隆，预防原发恶性疾病复发[15]–[20]。Tian等[19]研究结果显示亲缘单倍体造血干细胞移植后3个月CD8^+^T淋巴细胞恢复水平较高的患者疾病复发率更低，总生存明显改善。CD8^+^T淋巴细胞的早期重建依赖于效应记忆CD8^+^T淋巴细胞经非胸腺依赖途径扩增[20]，重建速度可能会受到移植后患者外周血中活性ATG的影响，所以ATG在预防GVHD的同时也可能抑制早期CD8^+^T淋巴细胞重建，带来潜在的复发风险[21]。目前的研究通常认为应用低剂量ATG的患者移植后复发风险较小[6],[8]，但Cho等研究显示在−3 d～−1 d使用2.5 mg/kg的rATG仍然有增加复发的风险。使用rATG组与不使用rATG组相比，rATG组的复发率有增加趋势（20.0％对9.3％，*P*＝0.055），并且在遗传学高危组患者中rATG组的复发率显著增加（29.6％对9.3％，*P*＝0.042）[22]。该研究中ATG剂量很小，却仍有较高的复发风险，可能与ATG给药时间接近移植物输注时间、移植后ATG作用过强相关。Lindemans等[10]在儿童无关供者脐带血移植的研究中发现，−9 d～−5 d使用ATG组免疫重建优于−5 d～0 d使用ATG组，也提示了给药时间接近移植物输注时间可能会延迟移植后免疫重建。Admiraal等[21]在成人无关供者移植的研究中发现移植后AUC过高会导致复发率增加和总生存率降低。上述研究说明ATG给药时间过于接近移植物输注时间，可能会导致患者免疫重建延迟，带来较高的复发风险。

本研究提示相比于以往ATG用药剂量的调整，调整ATG给药时间可能同样使患者受益。调整ATG用药时间使其远离移植物输注时间，可以在保证GVHD预防效果的同时，避免移植物回输后过高的ATG暴露量对供者来源T细胞的耗竭作用，可能更有利于患者移植后的免疫重建，降低复发风险，同时测量同胞全合患者移植后0～28 d血清活性ATG的AUC，可以在一定程度上预测患者免疫重建，AUC大于18 UE/ml·d，提示患者体内ATG代谢较慢，免疫重建受到不利影响，复发风险可能较高。

本研究存在以下局限：首先，相较于4d-rATG组，2d-rATG组的中位随访时间较短，该组患者存在进一步发生复发和死亡的可能；其次，本研究中进行了血清活性ATG浓度测量和淋巴细胞亚群分析的患者病例数较少，移植后活性ATG浓度与移植预后的关系尚需要多中心、大样本的研究进一步确定和验证。尽管如此，本研究以确凿的临床数据与ATG暴露、免疫重建监测数据，提出了rATG应用时间对恶性血液病患者MSD-HSCT预后可能存在的影响，有益于促进含rATG移植体系的优化。
